# Detection of *Rickettsia sibirica mongolitimonae* by Using Cutaneous Swab Samples and Quantitative PCR

**DOI:** 10.3201/eid2004.130575

**Published:** 2014-04

**Authors:** Julie Solary, Cristina Socolovschi, Camille Aubry, Philippe Brouqui, Didier Raoult, Philippe Parola

**Affiliations:** Aix-Marseille Université, Marseille, France

**Keywords:** tick, rickettsia, Rickettsia sibirica mongolitimonae, rickettsiosis, vector-borne diseases, bacteria, spotted fever group, cutaneous swab samples, quantitative PCR

**To the Editor:** Tick-borne rickettsioses are caused by the obligate intracellular bacteria spotted fever group (SFG) *Rickettsia* spp. These zoonoses are now recognized as emerging or reemerging human infections worldwide, with ≈15 new tick-borne rickettsial species or subspecies recognized as human pathogens during the 30 past years (*1*). New approaches have emerged in recent years to definitively identify the causative agents, including emerging pathogens. Using cutaneous swab specimens from patients for quantitative PCR (qPCR) testing rather than cutaneous biopsy specimens is a major innovation in the diagnosis of SFG rickettsioses (*2*–*4*). Using this approach, we report 1 of the few documented infections caused by *Rickettsia sibirica mongolitimonae*.

A 16-year-old boy with no medical history was admitted to the Department of Infectious diseases at University Hospital in Marseille on May 25, 2012, with a fever (40°C) and skin lesions on his lower right eyelid. He had been fishing 7 days earlier at a pond situated in southern France near Marseille (43°26′N, 5°6′E). He had been given amoxicillin/clavulanic acid by his family doctor and showed no improvement after 2 days. The only sign on physical examination was the presence of 2 eschars on his lower right eyelid, associated with right periorbital edema (Figure) and painful right-sided cervical lymphadenopathies. Results of standard laboratory tests were normal except for the C-reactive protein level (21 mg/L; reference value <10 mg/L). He reported that the black spots on his lower eyelid were most likely related to bites from ticks that he got while fishing. He removed the ticks the next day. Because a tick-borne rickettsiosis was suspected, oral empirical treatment with doxycycline (200 mg/daily) was started. The patient improved in 48 hours and remained well (Figure).

**Figure:**
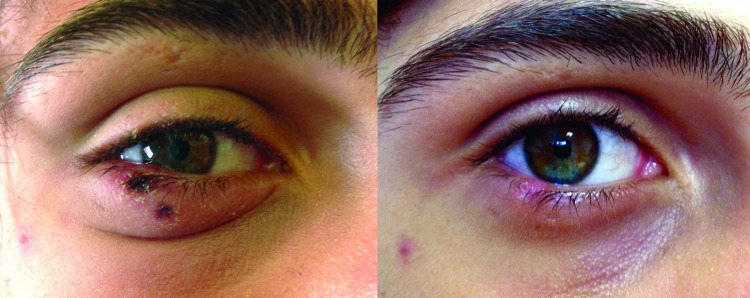
Palpebral eschars caused by *Rickettsia sibirica mongolitimonae* infection in a 16-year-old febrile boy with fever, southern France, spring, 2012 (left). He recovered after doxycycline treatment (right).

The first serologic test result for *Rickettsia* spp. was negative. Because of the location of the eschars, it was not possible to obtain biopsy specimens from them. Nevertheless, real-time qPCR that was performed on 2 eschar swab specimens showed positive results for *Rickettsia* spp in 24 hours. The specific qPCR test results were positive for *Rickettsia sibirica mongolitimonae* in both samples (*1*). 

Amplification and sequencing of a fragment of *ompA* gene on these samples showed 100% (533/533) identity with *R. sibirica mongolitimonae* HA-91 (RHU43796). Four days later, after doxycycline treatment, 1 additional swab specimen was positive by specific qPCR for *R. sibirica mongolitimonae.* The convalescent-phase serum specimen (obtained 14 days after admission) was positive by indirect immunofluorescence assay for rickettsial antigens against SFG, suggesting seroconversion.

*R. sibirica mongolitimonae* is an intracellular bacterium that was recognized as a human pathogen in 1996 (*1*). The inoculation eschar at the tick bite site is a hallmark of many tick-borne SPG rickettsioses. However, because lymphangitis was also observed in a few of the patients reported subsequently, *R. sibirica mongolitimonae* infection was named lymphangitis-associated rickettsiosis (*5*). To date, 24 cases have been reported in Europe (France, Spain, Portugal, Greece) and 3 in Africa (Egypt, Algeria, South Africa) (*6*,*7*). Vectors include ticks in the genus *Hyalomma* and also *Rhipicephalus pusillus*, a species of tick found on the European wild rabbit (also can be found on wild carnivorous animals, dogs, and domestic cats), which may bite humans (*7*). The life-threatening Mediterranean spotted fever caused by *R. conorii* peaks in the warmer months of July and August because of a heat-mediated increase in the aggressiveness and, therefore propensity to bite humans, of the brown dog tick vector, *R. sanguineus* (*8*). In contrast, *R. sibirica mongolitimonae* infection is more frequently reported in the spring (*7*).

The diagnosis of rickettsioses is most commonly based on serologic testing (*1*). However, serologic evidence of infection generally appears in the second and third weeks of illness, as in the case-patient described here. The use of molecular tools or cell culture on a skin biopsy specimen from an eschar is the best method of identifying *Rickettsia* spp. However, this invasive and painful procedure needs to be performed in sterile conditions with local anesthesia. Swabbing an eschar is easy and painless; the physician only needs a dry sterile swab that must be directed, while being rotated vigorously, to the base of the eschar, after the crust is removed (*4*). The sensitivity of this technique is comparable with that of rickettsial detection on skin biopsy samples by molecular tools. If the eschar lesion is dry, a wet compress, previously humidified with sterile water, should be placed on the inoculation eschar for 1 minute before swabbing, to increase the quantity of material swabbed. In addition, the crust eschar also can be used for rickettsial diagnosis. Because sufficient material can be obtained during swabbing, this test can be used by any practitioner at the patient’s bedside. As soon as the samples are sent to a laboratory with qPCR capability, results can be obtained quickly. In any case, when a physician is confronted with a patient with a fever and an eschar, doxycycline treatment should be initiated immediately because β-lactam antimicrobial drugs are inefficient for the treatment of rickettsioses (*9*).
